# Expansion planning of hybrid electrical and thermal systems using reconfiguration and adaptive bat algorithm

**DOI:** 10.1016/j.heliyon.2024.e36054

**Published:** 2024-08-11

**Authors:** Ali Reza Abbasi, Mahmoud Zadehbagheri

**Affiliations:** a Department of Electrical, Faculty of Engineering, Fasa University, Fasa, Iran; b Department of Electrical Engineering, Yasuj Branch, Islamic Azad University, Yasuj, Iran

**Keywords:** Expansion planning, Emission, Reliability, Self-adaptive learning bat algorithm (SALBA)

## Abstract

_ This study introduces a comprehensive model for the concurrent expansion planning of various energy systems and their associated equipment. The need to reduce network costs, emissions, losses, and feeder loading, as well as to enhance network reliability and voltage profile, mandates the utilization of proper multi-objective planning models that respect all network constraints. The introduced framework includes units for generating both electrical and thermal energies. The model leverages conventional expansion alternatives such as the installation of new lines, network reconfiguration, rewiring, and the addition of new thermal and electrical generating units to the network. Expansion planning involves determining the optimal time, location, and type of new installations to meet future energy demands while minimizing costs and emissions. Reconfiguration refers to altering the network topology to improve reliability and reduce losses. The proposed expansion planning is formulated as a discrete, nonlinear, and non-convex optimization problem, which is solved using the Self Adaptive Learning Bat Algorithm (SALBA). This algorithm improves convergence speed and increases the diversity of the search population, enhancing the likelihood of finding the global optimum. Numerical simulations of the proposed methodology on two modified standard IEEE test systems corroborate the efficacy and feasibility of the suggested approach. Key innovations include the comprehensive modeling for concurrent expansion planning, the use of an advanced optimization algorithm, and a focus on reducing costs, emissions, losses, and feeder loading while enhancing network reliability and voltage profile.

**Table:** Nomenclature

Symbol and description	
$C_{\text{Inv}}$	Cost of Investment
$C_{\text{Rel}.}$	Cost of reliability
$C_{\text{Loss}}$	Cost of energy loss
$C_{\text{Sub}}$	Cost of Generation
$N_{P}$	Number of power units
$N_{C}$	Number of CHP units
$N_{h}$	Number of heat units
$N_{L}$	Number of lines
$P_{j}^{h}$	Electric power equivalent thermal power at the jth bus
$P_{i}^{P}$	Net injected active power of the power unit at the ith bus
$P_{j}^{C}$	Net injected active power of the CHP unit at the jth bus
$N_{Cus}$	Total number of customers served
$\lambda_{i}$	Average failure rate of the ith component
$\rho_{i}$	Current compensation coefficient
$\left|I_{i}\right|$	Absolute current amplitude of the ith feeder
$\left|I_{new}^{i}\right|$	Current magnitude of ith branch after reconfiguration
$\left|I_{old}^{i}\right|$	Current magnitude of ith branch before reconfiguration
$U_{i}$	Annual outage time of the ith component
$\delta_{i}$	Angle of the voltage at the ith bus
$Y_{ij}$	Amplitude of admittance between the ith and jth buses
$\theta_{ij}$	Angle of branch between the ith and jth buses
${C_{i}}^{Elec.}$	Cost of a power unit that can be added at the ith bus
${C_{i}}^{CHP}$	Cost of a CHP unit that can be added at the ith bus
${C_{i}}^{Ther.}$	Cost of a heat unit that can be added at the ith bus
$C^{line}$	Cost of a circuit that can be added or upgraded between ith and jth bus
T	Planning horizon time
$P_{ij,\min}^{Line}$	Minimum active power between ith and jth bus
$P_{ij}^{Line}$	Expected value of active power between ith and jth bus
$P_{ij,\max}^{Line}$	Maximum active power between ith and jth bus
$P_{d}$	Electric power demand of system
$H_{C}^{j}$	Injected heat of the CHP unit at the ith bus
$H_{k}^{h}$	Injected heat of the heat unit at the kth bus
$H_{d}$	Thermal demand of the system
$H_{k}^{h\;\min}$	Minimum thermal outputs of the kth unit
$H_{k}^{h\;\max}$	Maximum thermal outputs of the kth unit
$\tau_{i},k_{j},\delta_{k}$	CO_2_ emissions coefficients
$\alpha_{i},\beta_{i},\gamma_{i},\zeta_{i},\lambda_{i}$	Emissions coefficients for the power-only units
$\theta_{j},\eta_{j},\pi_{k},\rho_{k}$	Emissions coefficients for the CHP units and heat-only units
$P_{j}^{C\;\min}\left(H_{j}^{C}\right)$	Minimum power limit of CHP unit j which are functions of generated heat ($H_{j}^{C}$)
$P_{j}^{C\;\max}\left(H_{j}^{C}\right)$	Maximum power limit of CHP unit j which are functions of generated heat ($H_{j}^{C}$)
$V_{\max}$	Maximum voltage magnitude
$V_{\min}$	Minimum voltage magnitude
$f_{i}\left(X\right)$	The ith objective function
$g_{i}\left(X\right)$	ith inequality constraint of MOP
$h_{i}\left(X\right)$	ith equality constraint of MOP
$N_{\text{eq}},N_{\text{ueq}\;}$	Number of equality and inequality constraints
$f_{i}^{\min}/f_{i}^{\max}$	Lowest/Highest limit of ith objective function
$P_{i}^{p\;\min}$	Minimum injected active power of the power unit at the ith bus
$P_{i}^{p\;\max}$	Maximum injected active power of the power unit at the ith bus
Swi	The ith sectionalizing switch
List of abbreviations	
EEP E	nergy Expansion Planning
CHP C	ombined Heat and Power
DFR D	istribution Feeder Reconfiguration
MOP M	ulti-objective Optimization Problem
SALBA S	elf-Adaptive Learning Bat Algorithm

## 1 Introduction

### 1.1 AProblem description and motivation

Increased efficiency due to the integration of combined thermal and electrical networks has motivated the implementation of these energy systems [1]. As a result, new operative tools need to be devised to address the Energy Expansion Planning (EEP) problem in systems containing combined thermal and electrical energy generating units. EEP involves answering four key questions about generating units and other network elements: the time, location, type, and method of integrating an element into the system over the entire planning horizon [2]. This integration must respect all network limits and satisfy the total load demand. Moreover, recent concerns related to fossil fuel resources, such as greenhouse gas emissions, suggest that planning goals should include the reduction of SO_2_, CO_2_, and NOx emissions. The introduction of Renewable Energy Resources (RERs) has also raised optimism about decreasing total environmental emissions compared to conventional power plants [3]. Consequently, the secure integration of Distributed Generating (DG) units into the network, considering economic and environmental concerns, has gained significant attention. Among various types of DGs, this paper focuses on three: power-only units, heat-only units, and Combined Heat and Power (CHP) units.

Through the expansion of energy units in future networks, enhancing system reliability should be considered, as reliability plays a vital role in keeping costs within a reasonable budget. As a result, this paper employs the Distribution Feeder Reconfiguration (DFR) strategy as a means of enhancing reliability. DFR aims to improve conditions by increasing reliability and reducing losses in the network through changes in network topology [4,5]. Within this context, the motivation of this research is to introduce a tool for the concurrent development of thermal and electrical energy systems. This tool facilitates decision-making for the installation of new units that generate electrical and thermal power, the addition of new lines, and the reconfiguration of the network over a specified timeframe. The goal is to ensure compliance with both equality and inequality constraints while reducing overall costs and emissions, and simultaneously enhancing the system's dependability.

### 1.2 BLiterature review

Earlier studies have primarily focused on enhancing the expansion of systems that utilize a single type of energy. For instance, Ref. [6] presents a development planning approach to analyze the distribution of heat-only energy demand and supply across Europe. By using a comprehensive thermal atlas, it identifies high-demand areas and aligns them with potential supply zones. Ref. [7] examines the potential risks associated with the expansion of liquefied natural gas infrastructure in Germany, highlighting that such expansion could increase dependency on gas and slow down the transition to renewable energies. Ref. [8] employs an adaptive robust optimization framework for planning the expansion of natural gas infrastructure in Europe, aiming to identify projects that can withstand fluctuations in gas demand and supply, thereby maintaining system stability. Ref. [9] introduces a multi-objective approach to planning the expansion of power generation and transmission infrastructure, incorporating capacitor bank allocation and demand response programs. Additionally, references [10,11] explore expansion planning in electricity distribution and transmission networks. The wide-ranging benefits of planning for energy expansion have garnered the focus of many investigators. Consequently, a variety of objectives have been specified. Numerous research efforts have been undertaken with the goal of reducing costs [12,13]. A novel optimization method based on squirrel and cat algorithms is introduced for optimal expansion planning of distribution systems. The approach aims to reduce costs and improve the efficiency of the power distribution system [12]. Several strategies have been formulated with the aim of improving reliability [14,15]. Ref. [14] evaluates the reliability and economic aspects of generation expansion planning by incorporating the variability of wind energy sources. It aims to optimize the balance between cost and reliability in power systems that integrate significant amounts of wind energy. Ref. [16] introduces an index to enhance the voltage stability via planning for network expansion. In addition to the aforementioned planning concerns, various studies have focused on the reduction of losses in power grids as an objective function, as indicated in Refs. [17,18]. A stochastic mixed-integer convex programming model is presented for long-term distribution system expansion planning, focusing on mitigating greenhouse gas emissions in Ref. [19]. The model aims to optimize the reinforcement of the distribution network while minimizing environmental impact. Moreover, the capability of DFR in making the network situation better through EEP is referenced in Ref. [20]. It should be noted that some studies solve the problem by considering only one of the mentioned criteria, while others use multiple objective functions. These studies have been developed for systems that are specific to a single type of energy, such as district heating, natural gas, and electricity systems.

The interdependence of electricity, gas, and heat networks has recently led to increased attention on multi-carrier energy systems. In Ref. [21], an optimal operation model is presented for coordinated multi-carrier energy hubs within integrated electricity and gas networks. The model aims to enhance efficiency and reliability by optimizing the interactions between these interconnected energy systems. Ref. [22] presents a mixed-integer linear optimization model for the integrated planning of municipal multi-energy carrier systems, focusing on achieving CO2 reduction targets. The model aims to design cost-effective energy systems that incorporate grid infrastructures and energy conversion units while minimizing greenhouse gas emissions. Ref. [23] presents a robust model for the expansion planning of integrated energy systems that include energy hubs, with a focus on ensuring reliability and efficiency in the planning process. Refs. [24,25] explores the combined expansion planning of gas and electricity networks, aiming to optimize the integration and coordination of both systems. Reference [26] reviews advanced models for the expansion planning of integrated power, natural gas, and hydrogen systems, with a focus on cost minimization and optimal facility placement.

Reference [27] discusses the coordinated expansion planning of natural gas and electric power systems, highlighting the importance of detailed representation for making optimal investment decisions. Refs. [26,27] both aim to improve system reliability and efficiency while managing uncertainties and reducing losses. Refs. [[28], [29], [30]] discuss optimal expansion planning of electricity and natural gas distribution systems with a focus on energy hubs and reconfiguration. Ref. [28] addresses the co-planning of reconfigurable electricity and natural gas distribution systems incorporating energy hubs to enhance operational performance. Ref. [29] focuses on resilience-constrained expansion planning of integrated power, gas, and heat distribution networks with reconfiguration to handle rare events. Ref. [30] presents a hierarchical robust expansion co-planning model for multi-energy distribution systems considering reconfiguration. Table 1 illustrates the classification of the mentioned literature review.

**Table 1:** Review of literature.

# Ref		Main concentrations in the objective function					Reconfiguration	Adding new			Rewiring	Objective Function		Optimization Method	
		Cost	Loss	Reliability	Feeder loading	Emission		Units	Load points	Line		Single	Multi		
[21]	✓	✓			✓		✓	✓	✓		✓			MILP	
[22]	✓		✓		✓		✓		✓			✓		MILP	
[23]	✓		✓				✓		✓		✓			MILP	
[24]	✓				✓		✓		✓		✓			MILP	
[25]	✓		✓		✓		✓	✓	✓			✓		MILP	
[26]	✓						✓		✓			✓		MILP	
[27]	✓		✓				✓		✓		✓			MILP	
[28]	✓		✓		✓	✓	✓		✓			✓		MIQP	
[29]	✓				✓	✓	✓		✓			✓		MILP	
[30]	✓		✓			✓	✓		✓			✓		MILP	
PM*	✓	✓	✓	✓	✓	✓	✓	✓	✓	✓		✓		SALBA	

### 1.3 CResearch gap

The main research gaps and shortcomings are listed below.

• Separate Consideration of Parameters: Previous studies have considered the parameters and objectives discussed in this paper, but they have done so separately rather than in a unified manner.
• Limited Diversity in Optimization Methods: Most studies have utilized MILP methods, showing little diversity in optimization approaches. Exploring other methods, such as evolutionary algorithms, could improve results.
• Focus on Cost and Reliability: The majority of studies concentrate on cost reduction and reliability enhancement. There is a need for more research on other aspects, such as system sustainability and flexibility.
• Inadequate Multi-Carrier Energy Studies: Studies on multi-carrier energy systems have not adequately addressed critical aspects such as reconfiguration, the creation of new load points for network expansion, rewiring, feeder loading, reliability, and environmental issues.
• Integrated Model Requirement: There is a need for an integrated model that addresses the parallel expansion planning of energy networks, including both thermal and electrical energy systems.
• Insufficient Attention to Environmental Impacts: Some studies do not adequately address environmental impacts. Further investigation in this area could contribute to the development of more sustainable systems.

### 1.4 DProcedure

As explained previously, the suggested EEP is a sophisticated problem with multiple objectives, requiring strong and robust algorithms to determine the best overall solution. For this reason, an improved Bat Algorithm (BA) is used for solving the suggested EEP. The BA emulates the bats behavior in chasing prey or searching for food. This algorithm is conceptually straightforward, making its implementation easy. However, the initial version of the algorithm is limited by the homogeneity of its population. To address this, various self-adjusting enhancement measures were incorporated into the original algorithm to improve its convergence speed and expand the diversity of its search population. Distributing the population throughout the entire search area increases the likelihood of discovering the global optimum. The strength and effectiveness of the proposed method were demonstrated by testing it on two distinct IEEE standard test systems.

### 1.5 Contributions

The innovations and main highlight of the paper can be summarized as follows.

• Comprehensive Modeling for Concurrent Expansion Planning of Various Energy Systems: This paper introduces an all-encompassing model for the concurrent expansion planning of various energy systems and their associated equipment. The model includes units for generating both electrical and thermal energies and utilizes conventional expansion alternatives like the installation of new lines, network reconfiguration, rewiring, and adding new thermal and electrical generating units and new load points to the network.
• Use of Modified Advanced Optimization Algorithm: The expansion planning problem is formulated as a discrete, nonlinear, and non-convex optimization problem, solved using the *Self Adaptive Learning* Bat Algorithm (SALBA). This algorithm improves convergence speed and increases the diversity of the search population, enhancing the likelihood of finding the global optimum.
• Focus on Reducing Costs, Emissions, Losses, and Feeder Loading: The proposed model aims to reduce network costs, emissions, losses, and feeder loading while enhancing network reliability and voltage profile.
• Utilization of Distribution Feeder Reconfiguration (DFR): The paper employs DFR as a strategy to improve system reliability by determining the best network topology to increase reliability indices. DFR provides better conditions in terms of increasing reliability and reducing losses by changing the network topology.
• Validation of the Proposed Model: Numerical simulations of the proposed methodology on two modified standard IEEE test systems corroborate the efficacy and feasibility of the suggested approach.

These innovations contribute to a comprehensive and efficient approach to energy system expansion planning, addressing both economic and environmental aspects.

### 1.6 Paper organization

The subsequent sections of the paper are structured as follows: Section 2 formulates the objective functions and constraints relevant to the issue at hand. Section 3 discusses the capability of DFR in improving reliability indices. Sections 4, 5 describe the original BA with proposed modifications and the application procedure, respectively. Section 6 delineates the simulated test systems, accompanied by the numerical outcomes and their corresponding discussions. The final section summarizes the key points and contributions of the paper.

## 2 Problem formulation

### 2.1 AMathematical representation of EEP

The proposed EEP is a non-linear, large-scale optimization problem characterized by non-convex and non-smooth solutions. The problem exhibits multiple modes and several local optima. As discussed earlier, the proposed EEP incorporates a range of objective functions, including minimizing total cost and minimizing emissions. These objectives are formulated as follows.

### 2.2 Minimizing total cost

Several sources impose costs on the network and must be considered when calculating the total network cost. These sources include system investment costs, the cost of the expected value of undistributed energy, energy loss costs, and the cost of energy imported from the upstream network. The total cost is the sum of these costs as follows:

(1) $$F_{Cost}=C_{\text{Inv}}+C_{\text{Rel}\;}+C_{\text{Loss}}+C_{\text{Sub}\;}$$

As obvious the total cost consists of four different terms that are explained in the following.

• *Investment Cost:* As mentioned previously, three types of generating units are considered: heat-only, power-only, and CHP units. The investment cost (C_Inv_) includes the costs associated with the installation of these units, as well as the costs related to the installation and/or reinforcement of lines, as follows:

N_C_, N_P_, and N_h_ represent the number of CHP, power-only, and heat-only units, with indices i, j, and k referring to them, respectively. Additionally, the amount of generated heat and power is denoted by H and P, and N_L_ represents the number of lines. The terms of the above formulation can be calculated using the following equations:

(4) $$\begin{array}{c} C_{i}\left(P_{i}^{P}\right)={C_{i}}^{Elec.}*T*P_{i}^{P}\left(3\right)\\ C_{j}\left(P_{j}^{C},H_{j}^{C}\right)={C_{i}}^{CHP}*T*\left(P_{j}^{C}+P_{j}^{h}\right)\\ C_{k}\left(H_{k}^{h}\right)={C_{i}}^{Ther.}*T*P_{k}^{h}\left(5\right)\\ C_{L}\left(L_{ij}\right)=C^{line}*n_{ij}*L_{ij}\left(6\right) \end{array}$$

• *Cost of Expected Value of Non-Distributed Energy*: ECOST is considered the system reliability index to specify the satisfactory level of dependability for consumers. It determines the economic evaluation of redundancy allocation and network reinforcement, as well as the identification of weak points in a system. Once the weak points in the system are detected, the operator should devise proper operation policies and maintenance schedules. Calculating ECOST requires evaluating load-point reliability indices. These indices consist of average outage time, annual system outage time, and average failure rate, which are computed as follows:

(7) $$r_{S}=\frac{U_{s}}{\lambda_{S}};U_{S}=\sum_{i}\lambda_{i}r_{i};\lambda_{S}=\sum_{i}\lambda_{i}$$

**Fig. 1:**
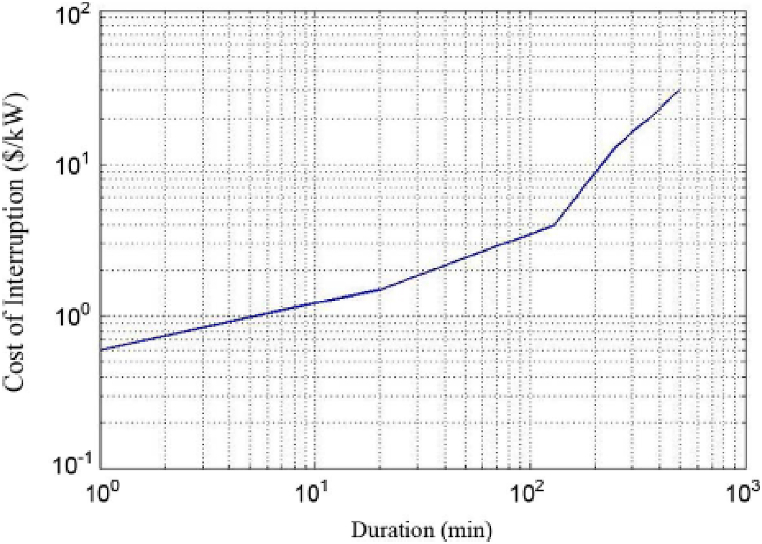
Typical CCDF.

For the ith bus, the calculation of ECOST is as follows [20]:

(8) $$ECOST_{i}=L_{i}C_{i}\lambda_{i}$$

where C_i_ denotes the interruption cost, which is determined by a nonlinear function based on the duration of the interruption. The C_i_ value, related to the time of interruption, is determined using the CCDF. To assess the costs associated with consumer interruptions, it is necessary to compute the system's load-point reliability indices for each switching configuration separately. In this case, a standard CCDF is employed to convert the energy losses experienced by customers into their monetary equivalents. Fig. 1 shows a typical CCDF [31].

For *n*_*bus*_ load points in the distribution feeder, the calculation of total ECOST is as follows:

(9) $$C_{\text{Re}l}=\sum_{i=1}^{nbus}ECOST_{i}=\sum_{i=1}^{nbus}L_{i}C_{i}\lambda_{i}$$

It is noted that CCDF specifies the value of bus interruption cost [31].

• *Cost of Energy loss:* To emphasize the importance of reducing energy loss in the system, a cost corresponding to its quantity is assigned according to the equation below:

(10) $$C_{Loss}=T\times C_{Ploss}\times \sum_{i=1}^{Nbr}R_{i}{\left|I_{i}\right|}^{2}$$

In which resistance and current of *i*th branch are denoted by *R*_*i*_ and |*I*_*i*_| respectively and *N*_*br*_ shows number of branches.

• *Cost of Generation:* C_sub_ represents the yearly cost of imported energy. The network cost can be ascertained in the *following* way:

(11) $$C_{sub}=price\times P_{sub}\times T$$

(12) $$P_{sub}=\sum_{j=1}^{n}\left|V_{sub}\right|\left|V_{j}\right|\left|Y_{sub,j}\right|\cos\left(\theta_{sub,j}-\delta_{sub}-\delta_{j}\right)$$

#### 2.2.1 Minimizing emission

This paper considers three sources of emissions: NOx, SO2, and CO2. The first two gas emissions are functions of the output power of the units. For power-only units, the function is exponential, while for heat-only and CHP units, it is linear, as follows:

(13) $$Em_{TypeofDG}^{No_{x,}So_{2}}=\left(\sum_{i=1}^{Np}\{10^{-2}\left(\alpha_{t}+\beta_{t}P_{t}+\gamma_{i}P_{i}^{2}\right)+\zeta_{i}e^{\left(\lambda_{i},P_{i}\right)}\}+\sum_{j=1}^{Nc}\left\{\left(\theta_{j}+\eta_{j}\right)O_{j}\right\}+\sum_{k=1}^{Nh}\left\{\left(\pi_{K}+\rho_{K}\right)TH_{K}\right\}\right)*T$$

In the above formulation, the output power of CHP and power-only units are denoted by *P*_*t*_ and *O*_*j*_, respectively. The emission factors for units that only generate power are represented by *α*_*i*_, *β*_*i*_, *γ*_*i*_, *ζ*_*i*_, and *λ*_*i*_. The emission coefficients for CHP units and heat-only units are denoted by *θ*_*j*_, *η*_*j*_ and *π*_*k*_, and *ρ*_*k*_, respectively. Similarly, CO2 emissions are represented by a linear function of the output power of the units as follows:

(14) $$Em_{TypeofDG}^{CO_{2}}=\left(\sum_{i=1}^{Np}\tau_{i}P_{i}+\sum_{i=1}^{Nc}k_{j}O_{j}+\sum_{k=1}^{Nh}\sigma_{k}TH_{k}\right)*T$$

CO_2_ emissions coefficients are shown by *τ*_*i*_, *k*_*j*_, *σ*_*k*_. The average emissions due to the power plants of the main grid (Em_grid_) are calculated as follows:

(15) $$Em_{grid}=\left(E_{No_{X}}^{grid}+E_{So_{2}}^{grid}+E_{CO_{2}}^{grid}\right)*P_{sub}$$

where $E_{No_{X}}^{grid}$, $E_{So_{2}}^{grid}$ and $E_{CO_{2}}^{grid}$ represent the average rates of NOx, SO2, and CO2 emissions of all committed power plants. The total emission of the network is then calculated by summing the above functions as follows:

(15a) $$F_{Emission}=Em_{TypeofDG}^{No_{x,}So_{2}}+Em_{TypeofDG}^{CO_{2}}+Em_{grid}$$

• *Constraints*

There are both equality and inequality constrains in the proposed formulation that need to be respected during EEP.

#### 2.2.2 Equality constraints

• Preserving radial structure of Distribution system

Generally, distribution networks are configured in a radial pattern, which offers advantages such as robust protection, ease of design, and affordability. Therefore, it is crucial to maintain the radial structure of these distribution systems. During optimization, if a loop emerges, a sectionalizing switch within the loop is activated to preserve the system's radiality. In this research, the 'depth-first search algorithm' is employed to identify any loops present in the system [32]. After identifying a loop, a branch within that loop is selected and opened at random. Subsequently, the 'depth-first search algorithm' is applied repeatedly until the network achieves a fully radial state.

• Power flow constraints:

(16) $$\begin{array}{c} P_{K}=V_{K}\sum_{j\in N}V_{j}\left(g_{jk}\;\cos\;\theta_{jk}+b_{jk}\;\sin\;\theta_{jk}\right) \end{array}$$

(17) $$Q_{K}=V_{K}\sum_{j\in N}V_{j}\left(g_{jk}\;\sin\;\theta_{jk}-b_{jk}\;\cos\;\theta_{jk}\right)$$

In these equations, the conductance and susceptance between the *j*th and *k*th buses are denoted by *g*_*jk*_ and *b*_*jk*_ respectively and the impedance angle of the respective branch is shown by *θ*_*jk*_.

#### 2.2.3 Inequality constraints

• Limits on heat production and consumption:

(18) $$\sum_{j=1}^{N_{C}}H_{C}^{j}+\sum_{k=1}^{N_{h}}H_{k}^{h}\geq H_{d}$$

*H*_*d*_ signifies the system's demand for heat.

• Limits on capacity of conventional units

(19) $$P_{i}^{p\;\min}\leq P_{i}^{p}\leq P_{i}^{p\;\max}i=1,\ldots ,N_{P}$$

Here *P*_*i*_^*pmin*^ and *P*_*i*_^*pmax*^ show the min and max output powers of the *i*th unit.

• Limits on capacity of CHP units:

(20) $$P_{j}^{C\;\min}\left(H_{j}^{C}\right)\leq P_{j}^{C}\leq P_{j}^{C\;\max}\left(H_{j}^{C}\right)j=1,\ldots ,N_{C}$$

(21) $$H_{j}^{C\;\min}\left(P_{j}^{C}\right)\leq H_{j}^{C}\leq H_{j}^{C\;\max}\left(P_{j}^{C}\right)j=1,\ldots ,N_{C}$$

Limits on *generation* of heat units:

(22) $$H_{k}^{h\;\min}\leq H_{k}^{h}\leq H_{k}^{h\;\max}k=1,\ldots ,N_{h}$$

*H*_*k*_^*hmin*^*and H*_*k*_^*hmax*^ show the lower and upper bounds of thermal outputs for the *k*th unit respectively.

• Limits on bus voltage:

(23) $$V_{\min}\leq V_{k}\leq V_{\max}k=1,2,\ldots ,N_{bus}$$

*V*_*k*_ is the voltage magnitude at the *k*th bus. *V*_min_ and *V*_max_ represent the lower and upper bounds on permissible bus voltages respectively.

• Limits on Line loading:

(24) $$PF_{k}\leq PF_{k}^{\max}$$

here *PF*_*k*_ and *PF*_*K*_^*max*^ are the amount of power flow in the *k*th branch and its maximum level.

## 3 Effect of Dfr on reliability

An analysis of failure statistics reveals that a significant number of interruptions in distribution systems are due to overhead and underground cable failures [33]. The resistive ohmic loss in underground cables generates heat, leading to insulation failure, which is primarily made of cross-linked ethylene-propylene or polyethylene rubber [34]. This failure occurs when the generated heat exceeds the tolerable levels defined for the cable. The temperature increases as the ohmic loss rises, which is a direct function of the square of the current magnitude through the cable. As the temperature in the cable rises, moisture can infiltrate the cable, causing damage to the dielectric insulation. The sensitivity of these insulations to moisture is the primary cause of breakdowns under overload conditions [33]. When the current flow through the cable increases and the temperature rises in overhead lines, the ground clearance is reduced. This reduction in ground clearance leads to increased electrical breakdowns and a higher failure rate [35]. All these conditions result in decreased system reliability. DFR has been shown to be capable of changing the flow and amount of current through the feeders of a system by opening or closing some remote switches located in the network. DFR alters the flow of power across feeders at almost no cost. It also provides the optimal structure for the system to minimize active loss. Reductions in active loss lead to decreases in the system's maximum loadability. Consequently, DFR can effectively enhance the system's reliability by reducing the current in overloaded lines and decreasing losses.

Altogether, DFR can be implemented as a strategy to reduce the failure rate and subsequently enhance reliability. To represent the mathematical formulation of the problem, the initial failure rate of a system line prior to reconfiguration can be indicated as *λ*_*i*_^*init*^, and the optimal failure rate achieved by complete active/reactive current compensation can be represented as *λ*_*i*_^*best*^. The current failure rate is computed as follows [36]:

(25) $$\lambda_{i}^{new}=\rho_{i}\left(\lambda_{i}^{init}-\lambda_{i}^{best}\right)+\lambda_{i}^{best};\rho_{i}=\frac{\left|I_{new}^{i}\right|}{\left|I_{old}^{i}\right|}$$

It should be particularly noted that the aforementioned method for reducing failure rates is generally most beneficial for feeders whose current levels are near their rated capacities. Therefore, feeders with low currents were ignored while performing this strategy. The criteria for selecting lines for this strategy rely on their electrical current; thus, only lines that carry a current greater than 80 % of their nominal capacity are chosen.

## 4 Optimization procedure

The basics of multi-objective optimization are discussed here, and an interactive fuzzy satisfying approach is introduced to solve the suggested EEP. In this method, the objective functions are converted into a min-max problem and solved as a single objective problem. Additionally, the proposed evolutionary algorithms are explained at the end.

### 4.1 AMulti-objective optimization framework

In a Multi-objective Optimization Problem (MOP), multiple objectives compete with one another. The problem should be solved to find a solution that satisfies all the objectives to some extent. A typical MOP problem can be represented as follows:

(26) $$\begin{array}{c} \min\;F={\left[f_{1}\left(X\right),f_{2}\left(X\right),\ldots ,f_{n}\left(X\right)\right]}^{T}\\ s.t\text{.}\\ g_{i}\left(X\right)≺0i=1,2,\ldots ,N_{ueq}\\ h_{i}\left(X\right)=0i=1,2,\ldots ,N_{eq} \end{array}$$

**Fig. 2:**
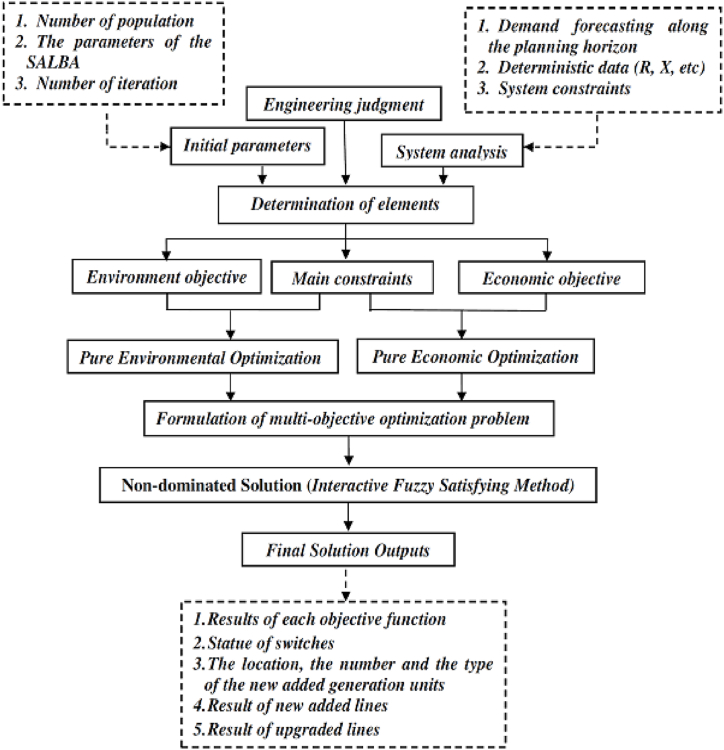
Multi-objective optimization planning model.

Here, *fi(X)* denotes the *i*th objective function, while equality and inequality constraints are represented by *g*_*i*_*(X)* and *h*_*i*_*(X)*, respectively*.* The number of equality constraints is *N*_*eq*_ and the number of inequality constraints is *N*_*ueq*_. Fig. 2 illustrates the flow chart of the multi-objective optimization model.

### 4.2 BFuzzy Modelling for normalization of objective functions

To solve the multi-objective problem, the objective functions are normalized using a fuzzy technique. This method converts the impreciseness of the objectives into exact values through fuzzy sets and a membership function (*μ*_*fi*_), as shown in Eq. (28). The membership function, which takes values between 0 and 1, depicts the level of adaptability of each objective. Due to the varying characteristics and behaviors of the examined objective functions, a fuzzy membership function is used to express all the objective functions uniformly [42].

(27) $$\mu_{fi}\left(X\right)=\left\{ \begin{array}{cc} 0 & f_{i}≻f_{i}^{\max}\\ \frac{f_{i}^{\max}-f_{i}}{f_{i}^{\max}-f_{i}^{\min}} & f_{i}^{\min}\leq f_{i}\left(X\right)\leq f_{i}^{\max}\\ 1 & f_{i}≺f_{i}^{\min} \end{array}\right.$$

The minimum acceptable level (*f*_*i*_^*min*^) and the maximum level (*f*_*i*_^*max*^) of the *i*th objective function are determined through the individual optimization of that function.

### 4.3 CInteractive fuzzy satisfying technique

The preferred solution is extracted from the collection of optimal solutions using an interactive fuzzy satisfaction technique [37]. First, separate membership functions are assigned to all objectives, and desired levels of preference (reference membership values) are specified for each function. The reference value (*μ*_*ri*_) determines the achievement level of each objective and takes a value between 0 and 1. If an objective is more important, higher values of *μ*_*ri*_ are assigned to it.

Once the membership functions and reference values are set, the following min-max problem is solved to obtain the desired solution:

(28) $$\min_{X\in \varphi }\left(\max_{i}\left|\mu_{ri}-\mu_{fi}\left(X\right)\right|\right)$$

Ω is the set of non-inferior solutions. It should be noted that if there is no biased preference among the objectives, the following problem will be solved instead:

(29) $$\max_{P\in \Omega }\left(\min_{n=1,\ldots ,n0}\left|\mu_{fn}\right|\right)$$

### 4.4 Self-adaptive Learning Bat algorithm

#### 4.4.1 Original BA

Bats locate nourishment or prey using echolocation. Echolocation involves determining the location of objects by interpreting their responses to specific subsonic signals. The bat dispatches a signal in the surrounding environment and remains on the lookout for the echoed signal to return from its prey or food. This concept has been modeled to create a metaheuristic optimization technique known as the Bat Algorithm (BA) [38].

To emulate the algorithm, it is important to understand that every bat uses echolocation to measure distances. A bat located at position X_m_ or moving at a velocity of Vel_m_ emits a signal. This signal has a frequency, denoted as f_min_, a wavelength, represented by λ, and an amplitude, which indicates its loudness, denoted as A_0_. The echoes returned from various objects differ, allowing bats to distinguish between prey and other objects. Adjustments are made to the wavelength and its emission rate, denoted as r. Simultaneously, the amplitude of the signal for the ith bat, indicated by A_i_, is gradually reduced from a high initial value, A_0_, to the minimum value, A_min_. The process begins with the creation of an initial set of bats, randomly distributed within the search domain. The evolutionary process involves modifying the positions of the bats based on subsequent adjustments.

(30) $$\begin{array}{c} \text{Ve}l_{m}^{k+1}=\text{Ve}l_{m}^{k}+\left(\text{Gbes}t^{k}‐X_{m}^{k}\right)f_{m}m=1,\ldots ,N_{\text{Bat}}\\ X_{m}^{k+1}=X_{m}^{k}+\text{Ve}l_{m}^{k+1}m=1,\ldots ,N_{\text{Bat}} \end{array}$$

(31) $$f_{m}=f_{m}^{\min}+rand\left(.\right)\left(f_{m}^{\max}-f_{m}^{\min}\right)m=1,\ldots ,N_{Bat}$$

To imitate the random patterns of bat flight, a random number (*rand*) is generated and compared to *r*_*m*_^*k*^*.* If *rand* exceeds this threshold, a new solution is created near the bat's current location in the following way:

(32) $$X_{new}=X_{old}+\varepsilon A^{k}$$

After the bats' locations are improved through the changes mentioned earlier, a new random entity, X_new_, is created if its signal rate, r_i_, exceeds a randomly determined number. This new solution will be added to the existing population if it meets the following condition.

(33) $$\left[rand≺A_{i}\right]\&\left[f\left(X_{i}\right)≺f\left(X_{gbest}\right)\right]$$

As previously stated, the amplitude values of signals produced by bats diminish progressively, as expressed by the following formulation:

(35) $$\begin{array}{c} A_{i}^{k+1}=\alpha A_{i}^{k}\left(34\right)\\ r_{i}^{k+1}=r_{i}^{0}\left[1-\exp\left(-\gamma k\right)\right] \end{array}$$

#### 4.4.2 Self-adaptive learning (SAL) mechanism

The initial version of the BA has certain limitations, such as the risk of getting trapped in local optima and a slow convergence rate towards the best solution. Adaptability is a key feature of any heuristic optimization technique. Therefore, a modification phase is added to the original BA to enhance this feature. The SAL method increases diversity within the population, helping the algorithm escape local optima. During this phase, each entity encounters four different modification strategies, each with varying probabilities, and must adaptively choose the most suitable modification based on its probability. The likelihood of selecting a strategy depends on how well it optimizes the fitness function. The strategies are as follows:

*Mutation strategy 1*: This approach is intended to explore various directions near the optimal solution, thereby enhancing the algorithm's overall search efficiency. Using the optimal solution, denoted as *Gbest*^k^, a new entity is created as follows:

(36) $$\begin{array}{c} X_{m,1}^{k}=X_{m}^{k}+round\left(1+rand\left(.\right)\right)\left(Gbest^{k}-Mean^{k}\right)\\ m=1,\ldots ,N_{1}^{k} \end{array}$$

*Mutation strategy 2*: This approach, inspired by natural processes, aims to enhance the algorithm's local search capabilities, leading to a faster. The core idea of this method is to seek the optimal solution while avoiding the least favorable one. This is achieved by creating a unique individual as follows:

(37) $$X_{m,2}^{k}=X_{r1}^{k}+rand\left(.\right)\left(Gbest^{k}-Worst^{k}\right)m=1,\ldots ,N_{2}^{k}$$

*Mutation strategy 3:* The third method enhances the variety among the bat population through the creation of an individual based on mutation in the following way:

(38) $$\begin{array}{c} X_{m,3}^{k}=X_{r1}^{k}+rand{\left(.\right)}_{1}\left(X_{r2}^{k}-X_{r3}^{k}\right)+rand{\left(.\right)}_{2}\left(X_{r4}^{k}-X_{r5}^{k}\right)\\ m=1,\ldots ,N_{3}^{k} \end{array}$$

*Mutation strategy 4:* Analogous considerations that applied to the former approach are pertinent to this one as well. In this method, the newly devised individual, which relies on mutation, is formulated as follows:

(39) $$\begin{array}{c} X_{m,4}^{k}=\left(\frac{X_{r1}^{k}+X_{r2}^{k}+X_{r3}^{k}}{3}\right)+\left(R_{1}-R_{2}\right)\left(X_{r2}^{k}-X_{r1}^{k}\right)+\left(R_{3}-R_{2}\right)\\ \left(X_{r2}^{k}-X_{r3}^{k}\right)+\left(R_{1}-R_{3}\right)\left(X_{r3}^{k}-X_{r1}^{k}\right)m=1,\ldots ,N_{4}^{k} \end{array}$$

where $R_{1}=F\left(X_{r1}^{K}\right)/R^{\prime },R_{2}==F\left(X_{r2}^{K}\right)/R^{\prime },R_{3}==F\left(X_{r3}^{K}\right)/R^{\prime },R^{\prime }=\left|F\left(X_{r1}^{K}\right)\right|+\left|F\left(X_{r2}^{K}\right)\right|+\left|F\left(X_{r3}^{K}\right)\right|,N_{1}^{it},N_{2}^{it},N_{3}^{it}\text{,}$ and $N_{4}^{it}$ represent the quantities of bats that migrate in accordance with mutations 1, 2, 3, and 4 during the k-th iteration. Vectors $X_{r1}^{k},X_{r2}^{k},X_{r3}^{k},X_{r4}^{k},andX_{r5}^{k}$ are selected at random from the population. The strategy that has a greater likelihood will be chosen for execution from the given options If the new test individuals created by this strategy improve the fitness function, they will replace the *i*th bat. Initially, each strategy has an equal probability of 0.25. This probability is updated after LP iterations based on the success rate of the trial solutions generated by the *a*th mutation strategy (*SR*_*a*_^*k*^), as described below:

(40) $$\mathrm{Pr}\;ob_{a}^{k}=\frac{SR_{a}^{k}}{\sum_{a=1}^{4}SR_{a}^{k}};a=1,\ldots ,4$$

The rate of success is determined by the *ns*_*a*_^*it*^ count of trial solutions yielded by the a-th mutation strategy and the *nf*_*a*_^*it*^ count of trials that did not succeed, as calculated in the subsequent manner:

(41) $$SR_{a}^{k}=\frac{\sum_{it=k-LP}^{k-1}ns_{a}^{it}}{\sum_{it=k-LP}^{k-1}\left(ns_{a}^{it}+nf_{a}^{it}\right)}+\delta ;a=1,\ldots ,4;k≻LP$$

The fixed parameter δ is established at 0.01 to prevent a zero value for the success rate. As per equation (40), the total probabilities for choosing strategies sum up to one. Every member of the bat group might execute the strategy deemed most likely using a Roulette Wheel Mechanism (RWM) during each cycle to attain the optimal solution. The procedure for selecting the method is outlined in the following description.

## 5 Application of optimization technique to Eep

To implement the suggested approach for the multi-objective EEP, follow these steps.

Step 1. Initialize the Algorithm:
• Define the input data and generate the initial bat population randomly. It is important to note that the bats represent feasible solutions for the problem.
• The required data includes:

Network Data: Sectionalizing switches, tie switches, nominal voltage, branch data, bus data, etc.
Reliability Data: Repair rate and failure rate of network components, etc.
Algorithm Parameters: Population size, termination criterion, adjusting parameters, etc.

Step 2. Evaluate All Objective Functions:
• Execute the power flow analysis by considering the decision variables and the constraints associated with the problem for each solution.
• This process determines the values of the objective functions and their corresponding membership functions.
Step 3. Apply the Interactive Fuzzy Satisfaction Technique:
• Implement the interactive fuzzy satisfaction technique across the entire population.
• Store the results in the repository.
Step 4. Identify the Best and Worst Solutions:
• Select the best and worst solutions from the population based on the evaluation criteria.
Step 5. Update the Bat Population:
• Move the bat population to new positions as described in section IV.D.1.
Step 6. Execute the Self-Adaptive Modification Phase:
• Perform the proposed self-adaptive modification phase as outlined in section IV.D.2.
Step 7. Update the Repository with New Non-Dominated Solutions:
• Use the new non-dominated solutions to update the repository.
• Check the repository size as described in Section IV.B–C.
Step 8. *Repeat the Process if Necessary:*
• If the termination criterion is not satisfied, return to step 4 and continue the process.

## 6 Numerical simulations

### 6.1 Case description

The proposed methodology is applied to two different modified IEEE test systems to demonstrate its feasibility and robustness. The reliability parameters are defined consistently for both cases and are explained in this section. The line with the highest impedance is estimated to have a failure rate of 0.4 failures per year, while the line with the lowest impedance has a failure rate of 0.1 failures per year. The failure rates for the other lines are calculated relative to these values, based on the length of each line [39]. Additionally, the required repair time for the lines is set to 6 h. A main breaker is installed in the main feeder, and a sectionalizer is planned to be placed at the beginning of each branch or line. The emission coefficients for the units are provided in Table 2.

**Table 2:** Emission coefficients.

Coefficient	*α*_*i*_	*β*_*i*_	*γ*_*i*_	*ζ*_*i*_	*η*_*j*_	*θ*_*j*_
Value	0.02543	−0.06047	0.05638	0.0005	0.0015	0.00015
Coefficient	*π*_*k*_	*ρ*_*k*_	*τ*_*i*_	*k*_*j*_	*σ*_*k*_	*-*
Value	0.008	0.001	0.28	0.2	0.4	–

Regarding the optimization techniques, the population size for SALBA is set to 30, and the maximum number of iterations is set to 50. Additionally, both *α*^*Bat*^ and *γ*^*Bat*^ are set to 0.9. The variable (*r*_*g*_) is assigned a random value, and *A*_*g*_ is set to 1. Furthermore, to confirm the validity of the results, they are benchmarked against three widely-used evolutionary algorithms: Honey Bee Mating Optimization (HBMO), Particle Swarm Optimization (PSO), and Genetic Algorithm (GA) [[39], [40], [41],^43^]. The settings for these algorithms are as follows.

• *PSO*: The cognitive and social parameters are both set to 2. The initial and final inertia weight factors are 0.9 and 0.4, respectively. The population size (N_pop_) is 30, and the maximum number of iterations (It_max_) is 50.
• *GA*: The maximum number of iterations (Itmax) is 50, the population size (N_pop_) is 30, the crossover rate is 0.3, and the mutation probability is 0.09.
• *HBMO*: The parameters S_max_, α^HBMO^, N_Worker_, N_Dreone_, N_Sperm_, and N_Brood_ are set to 1, 0.93, 10, 16, 15, and 15, respectively.

These parameters are consistently applied to all test systems. Additionally, the error criterion for the iterative method is set at 0.001. The simulations were carried out in a Matlab 7.4 environment on a Pentium P4, Core 2 Duo 2.2 GHz PC with 2 GB of RAM.

### 6.2 Case I

**Fig. 3:**
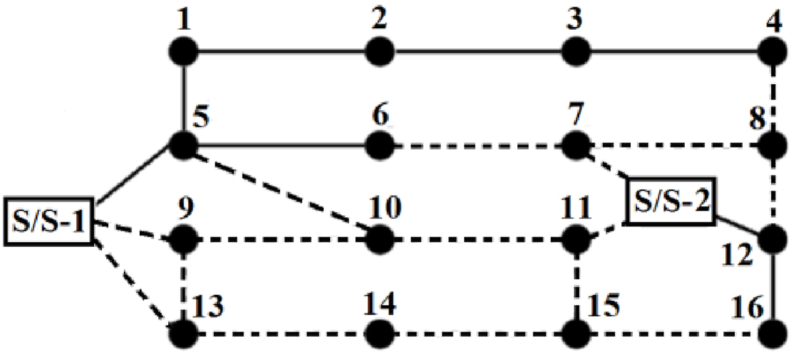
Schematic representation of the network with 18 nodes.

This 13.8 kV distribution network, as shown in Fig. 3, includes 16 load points, two distribution substations, 24 branches, and five looping branches [35]. Each branch is isolated from the network by an isolator switch. The network has five branches that can be upgraded, eight new load points, and 16 paths for installing additional load points. The original system does not include any thermal loads; therefore, arbitrary thermal loads are considered in this paper. The capacities of these thermal loads are 1 MW, 800 kW, and 650 kW, located at buses 2, 11, and 14, respectively.

Other parameters and specifications of the system are provided in Table 3. Table 4 shows the new connection points and load points. It is assumed that the cost of electrical and thermal units is not affected by their technology or type, so a standard cost is assigned to each unit. Units with a capacity of 1 MVA, which only supply power, can be installed at any node that handles loads. In contrast, the 1 MW CHP units and the 150 kW heating units can only be connected to nodes with a thermal demand, and their connection order is determined by a predefined priority. The optimal values for these parameters are obtained by running the HBMO, PSO, GA, and SALBA algorithms 50 times each.

**Table 3:**
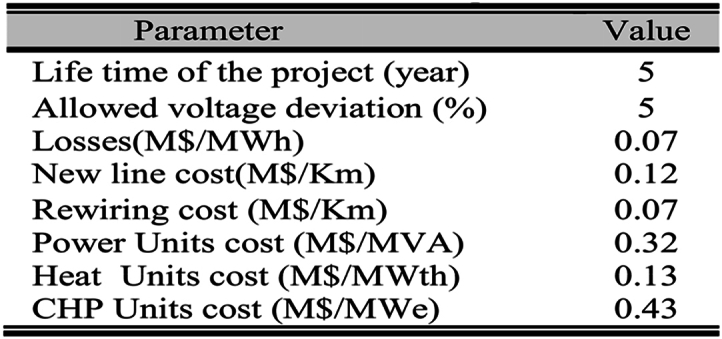
Technical/Cost parameters.

**Table 4:** New points of connection and load and their code.

Load Point	Connection Points	Load Point	Connection Points
7	S/S-2, 6, 8	11	S/S-2,10, 15
8	4, 8, 12	13	S/S-1, 9, 14
9	S/S-1, 10, 13	14	13, 15
10	5, 9, 11	15	11, 14, 16

**Fig. 4:**
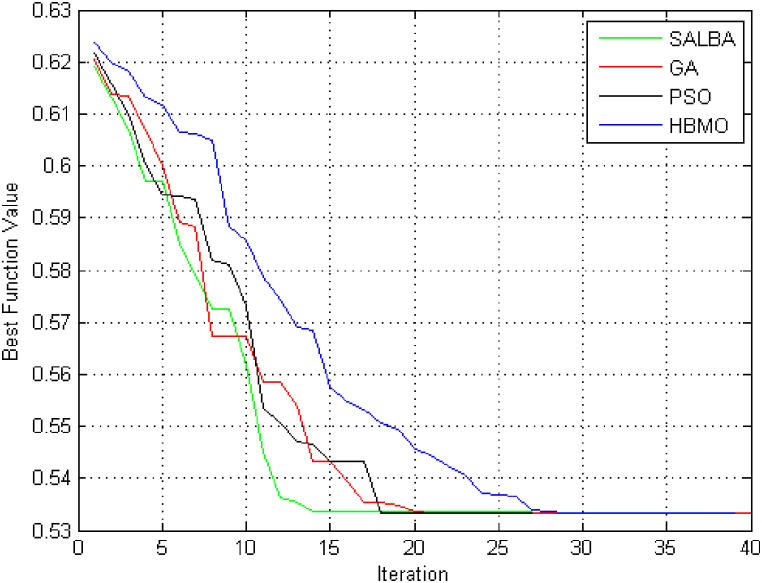
Evaluating the convergence trends for the objective function through ITLO, HBMO, PSO, and GA algorithms for 18-bus test system.

To demonstrate the effectiveness of the SALBA algorithm, a comparative display of the convergence plots for the objective function is presented. Fig. 4 shows that the algorithms require different numbers of iterations to achieve convergence.

The figure confirms the advantage of the proposed algorithm over the PSO, GA, and HBMO methods. Since the final results of these algorithms are equivalent, only the outcomes from the proposed method are detailed. The suggested EEP issue has been resolved, and the corresponding results are presented in Table 5, Table 6.

**Table 5:** Findings for every individual objective function.

Objective Function	Values
Cost	28271.52
Emission	7362.31

**Table 6:** Outcomes associated with the newly incorporated generation units.

Bus	No. of the units		
	Power units	Heat units	CHP units
2	0	0	2
10	2	0	0
11	0	2	1
14	1	1	1

**Fig. 5:**
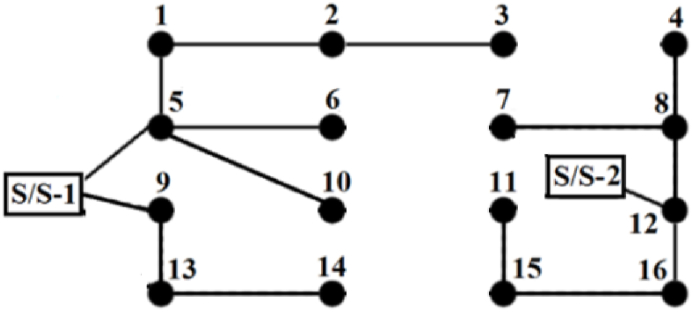
The best compromised solution for the reconfiguration network.

Table 5 presents the values of the objective functions, while Table 6 shows the locations, types, and numbers of newly installed generating units. Fig. 5 illustrates the single-line schematic of the distribution system, representing the optimal compromise solution.

In this figure, the branch labeled S/S-1-5 indicates that the branch between substation 1 and bus 5 needs to be rewired. Other branches that need rewiring are S/S-2-12 and 12–16. Additionally, the newly installed lines are S/S-1-9, 4–8, 5–10, 7–8, 8–12, 9–13, 11–15, 13–14, and 15–16. Switches are labeled according to the buses they connect to; for example, 'switch3-4′ refers to the switch located on the segment connecting buses 3 and 4, which needs to be opened to maintain the radial configuration of the network.

### 6.3 Case II

**Fig. 6:**
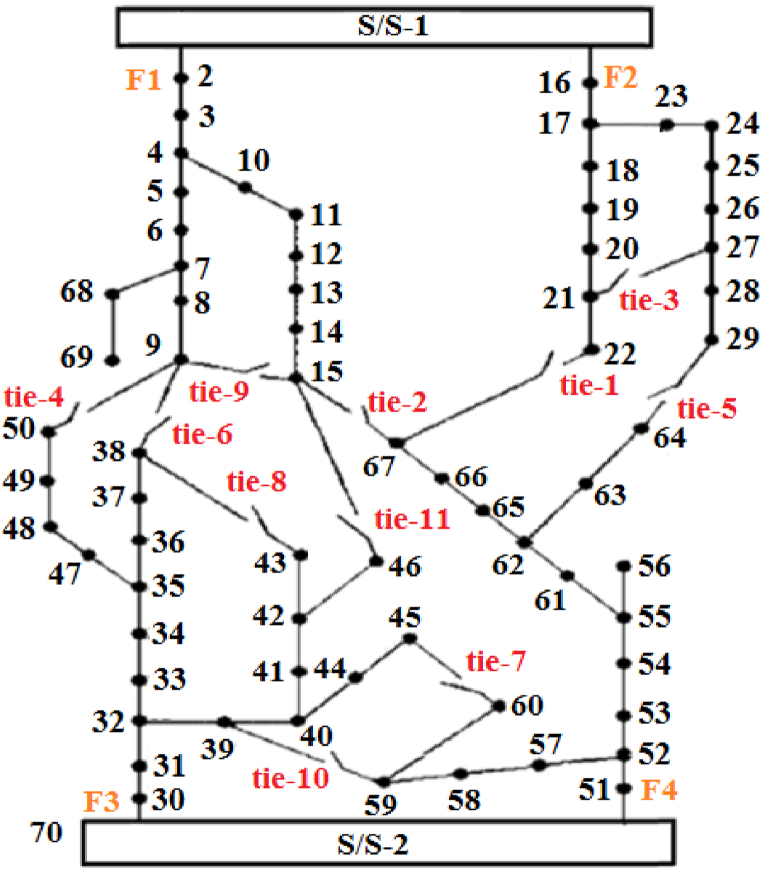
Schematic representation of the network with 70 nodes.

Fig. 6 [36] illustrates the single-line schematic of the secondary test setup. This setup consists of an 11 kV radial distribution network that includes two substations and four feeders, with a total of 70 nodes and 78 branches, including tie branches.

Similar to the previous case, arbitrary thermal loads are considered for this system as specified in Table 7. Additionally, there are 8 existing branch sections that can be upgraded, 3 new load points, and 9 paths for the installation of new branch sections, as detailed in Table 8.

**Table 7:** Information the thermal loads and the system.

Bus No.	9	27	44	57	62
Thermal load (kW)	200	250	150	300	250

**Table 8:** Information of new candidate branches.

No. of new branch	From	To	R(Ω)	X(Ω)
1	71	9	0.2	0.3
2	71	15	0.1	0.15
3	71	30	0.15	0.25
4	72	45	0.1	0.15
5	72	59	0.1	0.15
6	72	60	0.15	0.25
7	73	21	0.2	0.3
8	73	27	0.15	0.25
9	73	28	0.1	0.15

**Fig. 7:**
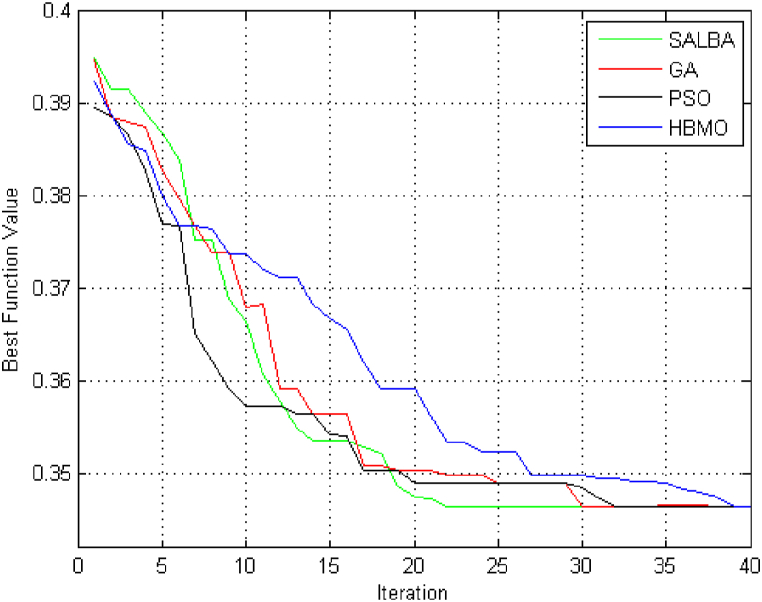
Evaluating the convergence trends for the objective function through ITLO, HBMO, PSO, and GA algorithms for 70-bus test system.

The selection of candidate locations for the installation of various units is based on the same criteria as the previous case. However, the maximum size of power units is 200 kW, the size of CHP units is 300 kW, and the capacity of heat units is 50 kW. Fig. 7 displays the convergence graphs for the objective function using the HBMO, PSO, GA, and SALBA algorithms. It shows that the proposed algorithm reaches the global optimum solution in just 22 iterations, while the other algorithms require over 30 iterations to converge to global solutions. In other words, SALBA achieves convergence approximately 1.4 times faster than the others. The figure clearly indicates that the SALBA algorithm is more effective for the EEP task than its counterparts, achieving convergence to the global optimum in fewer iterations, which is crucial in the computational operations of the power systems field. The capability of DFR is examined in this case by performing the proposed EEP. Analyzing the results given in Table 9, Table 10, Table 11 reveals the efficacy and superiority of SALBA. It is noted that the SALBA parameters were obtained by running the algorithm 100 times.

**Table 9:** Results of network reconfiguration and objective function.

Objective Functions Values		Open sectionalizing switches	Closed tie switches
Cost	Emission		
13442.97	2628.12	S48–49, S14-15,S27-28,S58–59, S42–48, S65-66	tie1, tie4, tie5, tie9, tie10 and tie11

**Table 10:** Result for new added units.

Bus	No. of units		
	Power Units	Heat Units	CHP units
9	1	0	1
27	0	1	1
40	2	0	0
44	0	0	1
54	1	0	0
57	1	2	1
62	0	1	1

**Table 11:** Result for new upgraded or added lines.

New added lines	Upgraded lines
(9, 71), (45, 72), (27, 73)	(22,66), (66,67)

**Fig. 8:**
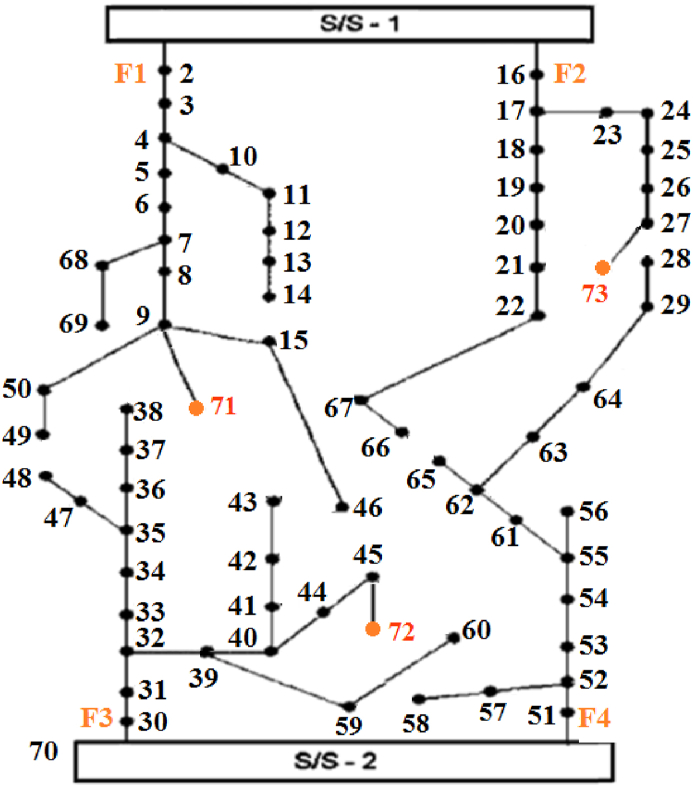
The best compromised solution of the distribution network.

SALBA has been successful in promptly reaching the best solutions for the objective functions. Fig. 8 illustrates the single-line representation of the test system after optimization. The objective function value associated with the positions of sectionalizing and tie switches is reported in Table 9.

According to this table, the following switches are opened: switch 14–15, switch 27–28, switch 42–48, switch 48–49, switch 58–59, and switch 65–66. The following switches are closed: tie1, tie4, tie5, tie9, tie10, and tie11. Altering the open/close status of these switches ensures the radial topology of the system. It is also noted that achieving an optimal value for the objective functions required the installation of new generating units, as detailed in Table 10.

Additionally, new lines 9–71, 45–72, and 27–73 are installed. To meet the problem requirements, lines 22–66 and 66–67 are rewired, as detailed in Table 11.

## 7 Conclusion

In this study, a comprehensive static Energy Expansion Planning (EEP) model was proposed to optimally plan the future expansion of multi-type energy generating systems, including both thermal and electrical systems, along with new planning alternatives. The considered generating units include power-only units, heat-only units, and Combined Heat and Power (CHP) units. The installation and advancement of network elements were fulfilled through the proposed EEP to guarantee a secure, cost-effective, and reliable configuration of the network capable of satisfying the predicted load demand.

The implementation of the proposed EEP model resulted in significant improvements across several key metrics. Firstly, there was a substantial reduction in overall network costs. The optimized expansion planning minimized the need for costly infrastructure investments while ensuring efficient energy distribution. Additionally, the integration of RERs and the optimized placement of new generating units led to a notable decrease in greenhouse gas emissions, successfully reducing SO_2_, CO_2_, and NOx emissions compared to conventional power plants. Furthermore, the reconfiguration of the network topology through DFR significantly reduced energy losses. The optimized network configuration ensured minimal transmission and distribution losses, enhancing overall system efficiency. The proposed methodology also improved network reliability by optimizing the placement and operation of generating units and reconfiguring the network. The reliability indices showed marked improvement, indicating a more robust and dependable energy system. Moreover, the optimized expansion planning and network reconfiguration resulted in a more stable voltage profile across the network, significantly enhancing the voltage stability index and ensuring consistent and reliable power delivery. The problem was solved using the SALBA algorithm, which takes advantage of adaptive modifications to diversify the searching population and achieve faster convergence to the global optimum solution. Application of the proposed modeling and solving methodology to two different test systems corroborated the veracity and robustness of the procedure. The methodology demonstrated its applicability to realistic large-scale systems and successfully minimized cost, minimized loss, enhanced reliability, and reduced emissions securely.

In conclusion, the results indicate that the proposed concurrent expansion planning model, combined with the advanced optimization capabilities of SALBA, provides a comprehensive and effective solution for the expansion of hybrid electrical and thermal systems. For future work, it is recommended that other aspects of EEP, such as dynamic modeling and stochastic planning of the system along with other resources such as batteries or demand respond, be analyzed.

## CRediT authorship contribution statement

**Ali Reza Abbasi:** Writing – review & editing, Writing – original draft, Visualization, Validation, Supervision, Software, Resources, Project administration, Methodology, Investigation, Funding acquisition, Formal analysis, Data curation, Conceptualization. **Mahmoud Zadehbagheri:** Writing – original draft, Visualization, Validation, Supervision, Software, Resources, Project administration, Methodology, Investigation, Funding acquisition, Formal analysis, Data curation, Conceptualization.

## Declaration of competing interest

The authors declare that they have no known competing financial interests or personal relationships that could have appeared to influence the work reported in this paper.
